# Feasibility of detecting atrophy relevant for disability and cognition in multiple sclerosis using 3D-FLAIR

**DOI:** 10.1007/s00415-023-11870-4

**Published:** 2023-07-19

**Authors:** Samantha Noteboom, D. R. van Nederpelt, A. Bajrami, B. Moraal, M. W. A. Caan, F. Barkhof, M. Calabrese, H. Vrenken, E. M. M. Strijbis, M. D. Steenwijk, M. M. Schoonheim

**Affiliations:** 1 MS Center Amsterdam, Anatomy and Neurosciences, Vrije Universiteit Amsterdam, Amsterdam Neuroscience, Amsterdam UMC location VUmc, Amsterdam, The Netherlands; 2 MS Center Amsterdam, Radiology and Nuclear Medicine, Vrije Universiteit Amsterdam, Amsterdam Neuroscience, Amsterdam UMC location VUmc, Amsterdam, The Netherlands; 3 Neurology B, Department of Neurosciences, Biomedicine and Movement Sciences, Regional Multiple Sclerosis Center, University of Verona, Verona, Italy; 4 Department of Biomedical Engineering and Physics, Amsterdam UMC location AMC, Amsterdam, The Netherlands; 5 Institutes of Healthcare Engineering and Neurology, University College London, London, United Kingdom; 6 MS Center Amsterdam, Neurology, Vrije Universiteit Amsterdam, Amsterdam Neuroscience, Amsterdam UMC location VUmc, Amsterdam, The Netherlands

**Keywords:** Multiple sclerosis, Atrophy, MRI, Disability, Cognition

## Abstract

**Background and objectives:**

Disability and cognitive impairment are known to be related to brain atrophy in multiple sclerosis (MS), but 3D-T1 imaging required for brain volumetrics is often unavailable in clinical protocols, unlike 3D-FLAIR. Here our aim was to investigate whether brain volumes derived from 3D-FLAIR images result in similar associations with disability and cognition in MS as do those derived from 3D-T1 images.

**Methods:**

3T-MRI scans of 329 MS patients and 76 healthy controls were included in this cross-sectional study. Brain volumes were derived using FreeSurfer on 3D-T1 and compared with brain volumes derived with SynthSeg and SAMSEG on 3D-FLAIR. Relative agreement was evaluated by calculating the intraclass correlation coefficient (ICC) of the 3D-T1 and 3D-FLAIR volumes. Consistency of relations with disability and average cognition was assessed using linear regression, while correcting for age and sex. The findings were corroborated in an independent validation cohort of 125 MS patients.

**Results:**

The ICC between volume measured with FreeSurfer and those measured on 3D-FLAIR for brain, ventricle, cortex, total deep gray matter and thalamus was above 0.74 for SAMSEG and above 0.91 for SynthSeg. Worse disability and lower average cognition were similarly associated with brain (adj. R^2^ = 0.24–0.27, *p* < 0.01; adj. R^2^ = 0.26–0.29, *p* < 0.001) ventricle (adj. R^2^ = 0.27–0.28, *p* < 0.001; adj. R^2^ = 0.19–0.20, *p* < 0.001) and deep gray matter volumes (adj. R^2^ = 0.24–0.28, *p* < 0.001; adj. R^2^ = 0.27–0.28, *p* < 0.001) determined with all methods, except for cortical volumes derived from 3D-FLAIR.

**Discussion:**

In this cross-sectional study, brain volumes derived from 3D-FLAIR and 3D-T1 show similar relationships to disability and cognitive dysfunction in MS, highlighting the potential of these techniques in clinical datasets.

**Supplementary Information:**

The online version contains supplementary material available at 10.1007/s00415-023-11870-4.

## Introduction

Accelerated brain tissue loss occurs from the earliest stages of multiple sclerosis (MS) and is associated with disability and cognitive impairment [1, 2]. Brain markers for neurodegeneration include brain volume measurements on magnetic resonance imaging (MRI). These markers are increasingly used in clinical trials, also as primary outcome measures [3–5]. As such trials have been successful and measures of atrophy have been shown to be predictive of long-term disease progression, there is now a need to implement measures for brain atrophy in clinical practice [6].

An important hurdle in this aim is the availability of appropriate MRI sequences in clinical scanning protocols. In general the clinical MRI protocols in MS include high-resolution 3D-FLAIR weighted sequences and post-contrast 3D-T1 sequences, because repeated scanning of lesions is mainly considered to be necessary for the identification and monitoring of inflammatory disease activity [7]. A high-resolution 3D-T1 image without contrast, traditionally considered the best sequence to identify neurodegeneration, is often not present in a clinical protocols due to time constraints.

To translate atrophy measurements to clinical datasets, there are increasing efforts to develop methods to measure brain volumes on clinical sequences. For example ventricular and thalamic volume have been measured on 2D-FLAIR scans, and total brain, grey matter (GM) and white matter (WM) volumes have been calculated on 3D-FLAIR [8–10]. However, not all of these methods are open source and they do not provide segmentation of cortical and deep GM structures separately.

Two new open source segmentation methods are Sequence Adaptive Multimodal SEGmentation (SAMSEG) [11] and SynthSeg [12]. Recent work has shown that both SAMSEG and SynthSeg are fast, reliable and reproducible on T1-weighted images [13, 14]. Since they are contrast-adaptive, both methods are promising candidates to evaluate brain atrophy on clinically acquired FLAIR-weighted images, but they still need validation in large MS datasets. Therefore, the objective of this study was to investigate the cross-sectional agreement between 3D-T1 and 3D-FLAIR brain volumetrics in MS using SAMSEG and SynthSeg segmentation methods. Furthermore, we investigated whether the methods on 3D-FLAIR were able to reach similar associations between brain volumes and clinical outcomes in MS as conventional 3D-T1. Lastly, results were externally validated in an independent dataset from a different center.

## Methods

### Participants

A total of 405 participants were retrospectively included from the Amsterdam MS cohort [15, 16] (The Netherlands) (Table 1). Subjects were selected based on the availability of a 3D-T1 weighted and 3D-FLAIR MRI scan with a voxel size smaller than 1.3 mm^3^. Included subjects comprised 329 MS patients with an established diagnosis of relapsing–remitting MS (RRMS, n = 242) or progressive MS (PMS, n = 87) and 76 healthy controls (HC). Approval was obtained from the local institutional ethics review board from both centers and written informed consent was provided by all participants.

**Table 1:** Information on demographics and disease related variables

	Amsterdam		Verona Validation cohort
	HC (n = 76)	MS (n = 329)	MS (n = 125)
Demographics			
Sex (female %)	57%	68%	74%
Age (years)	47.7 ± 9.8	48.0 ± 11.0	38.6 ± 9.8
Clinical characteristics			
Symptom duration (years)	–	14.5 ± 8.4	7.8 ± 6.6
EDSS	–	3.0 [2.5–4.5]	2.0 [1.0–3.0]
MS type (RRMS/PPMS/SPMS/UN)	–	242/36/51/–	112/6/6/1
Average cognition (Z-score)	− 0.03 ± 0.46	− 0.80 ± 0.94	− 0.30 ± 0.8
Normalized MRI volumes^a^			
Brain	0.736 ± 0.028	0.703 ± 0.044	0.727 ± 0.041
Ventricle	0.015 ± 0.006	0.023 ± 0.012	0.018 ± 0.008
Cortex	0.315 ± 0.017	0.308 ± 0.021	0.309 ± 0.02
DGM	0.031 ± 0.002	0.028 ± 0.003	0.031 ± 0.003
Thalamus	0.009 ± 0.001	0.008 ± 0.001	0.009 ± 0.001

Displayed are the mean and standard deviation of normally distributed continuous variables and the median and interquartile range of non-normally distributed data

*HC* healthy controls, *MS* multiple sclerosis, *CP* cognitively preserved, *CI* cognitively impaired, *EDSS* Expanded Disability Status Scale, *RRMS* relapsing remitting MS, *PPMS* primary progressive MS, *SPMS* secondary progressive MS, *UN* unknown

^a^MRI volumes were normalized by dividing volume by the segmentation based total intracranial volume (sbTIV)

### Clinical assessment

Clinical evaluation consisted of disability assessment by the Expanded Disability Status Scale (EDSS) and a the expanded Brief Repeatable Battery of Neuropsychological tests for cognitive assessment [17]. This BRB-N consists of the selective reminding test (SRT), spatial recall test (SPART), symbol digit modalities test (SDMT), word list generation test (WLG), Stroop color test, memory comparison test (MCT) and test concept shifting test (CST) [16]. Individual *Z*-scores were calculated for each test based on the means and standard deviations of the healthy controls, with correction for age, sex and education [18]. *Z*-scores from all tests were averaged to obtain the average cognition score.

### MRI acquisition

MRI data were acquired on a 3 Tesla GE Signa HDxt scanner (General Electric, Milwaukee, WI, USA) with an 8-channel head coil. The protocol included a 3D T1-weighted fast spoiled gradient echo (FSPGR) sequence (TR 7.8 ms, TE 3 ms, TI 450 ms, flip angle 12°, 1.0 mm sagittal slices, 0.94 × 0.94 mm^2^ in-plane resolution) and a 3D FLAIR sequence (TR 8000 ms, TE 125 ms, TI 2350 ms, 1.2 mm sagittal slices, 0.98 × 0.98 mm^2^ in-plane resolution).

### MRI image analysis

This study compared five different brain segmentation approaches. FreeSurfer on the lesion-filled 3D-T1 was used as reference segmentation. The volume output of FreeSurfer was compared to volume outputs of SAMSEG on 3D-FLAIR (SAMSEG_FLAIR_) and SynthSeg on 3D-FLAIR (SynthSeg_FLAIR_). In order to directly compare 3D-FLAIR vs. 3D-T1 within methods, both methods were also applied to 3D-T1 (SAMSEG_T1_, SynthSeg_T1_). Volumes of the brain, ventricle, cortex, and total deep gray matter (DGM, including summed bilateral volumes of the thalamus, caudate, putamen, pallidum, nucleus accumbens, hippocampus, and amygdala) were derived from the output of each segmentation method. These volumes were selected because of their clinical relevance in MS and their wide use as outcome measures in MS studies [19].

### FreeSurfer

The recon-all pipeline of FreeSurfer 7.1.1 [20] (http://surfer.nmr.mgh.harvard.edu/) was used to automatically perform whole-brain segmentation on 3D-T1 weighted images. This processing includes surface-based parcellation of the cortex [21] and segmentation of the subcortical white matter and DGM [22]. Since the presence of MS lesions affects the accuracy of FreeSurfer’s brain segmentation results, white matter lesions (WMLs) were filled on 3D-T1 prior to running FreeSurfer [23]. In short, WML were automatically segmented with the lesion prediction algorithm (LPA, SPM12) on 3D-FLAIR and filled on 3D-T1 with the SLF toolbox [24, 25].

### SAMSEG

SAMSEG is a Bayesian modelling algorithm within the FreeSurfer package (Samseg—Free Surfer Wiki (harvard.edu)), which allows segmentation of neuro-anatomical structures on any MRI contrast without the need for preprocessing [11]. 3D-FLAIRs were segmented with the MS-specific pipeline of SAMSEG (released in FreeSurfer 7.2), which is a dedicated extension of SAMSEG that simultaneously segments brain structures and WMLs (settings: 1 as *lesion mask pattern* and the default lesion threshold of 0.3). 3D-T1s were segmented with settings 0 as *lesion mask pattern* and the same lesion threshold of 0.3.

### SynthSeg

SynthSeg is a convolutional neural network (CNN) approach trained to segment brain structures on any MRI contrast and resolution, available as part of the FreeSurfer 7.3.2 package (SynthSeg—Free Surfer Wiki (harvard.edu) [12]. Similar to SAMSEG, SynthSeg does not require any preprocessing. While SynthSeg does not segment WMLs, the method has been trained to be robust to the presence of lesions. SynthSeg 2.0 was used to segment both native 3D-FLAIRs and 3D-T1s.

### Head size normalization

Normalization of brain volumes by head size is an important step when studying disease-driven neurodegeneration in a cross-sectional study design [26]. The default head size normalization method of FreeSurfer is the so-called “estimated total intracranial volume (eTIV)”, which has been found to be biased by total brain volume and requires manual quality control [27]. Therefore, FreeSurfer-based reference volumes were normalized by the segmentation-based intracranial volume (sbTIV) from the SAMSEG processing stream. sbTIV is proposed as a more robust alternative by FreeSurfer and is less sensitive to brain atrophy [28]. The volumes of all SAMSEG and SynthSeg-derived segmentations were normalized by dividing by the sbTIV of the corresponding pipeline.

### Statistical analysis

Agreement between FreeSurfer reference volumes on 3D-T1 and SAMSEG/SynthSeg derived raw volumes on 3D-FLAIR and 3D-T1 was evaluated by calculating intraclass correlation coefficients (ICCs) for consistency (single measures) with a 95% confidence interval (CI) in R statistical software (version 4.0.3; R Foundation for Statistical Computing, Vienna, Austria), to study potential systematic biases between methods. ICCs were calculated separately for HCs and MS. Agreement between head size estimates on 3D-FLAIR and 3D-T1 was analyzed with linear regression analysis with sbTIV on 3D-T1 as reference.

To evaluate to which extent FLAIR-based segmentation can detect differences between patients and controls as well as between different MS subtypes, normalized volumes were compared with analysis of covariance (ANCOVA, Pingouin 0.5.2 [29]) between HC vs. MS and RRMS vs. PMS, with age and sex as covariates. Effect sizes (partial η^2^) from group comparisons were calculated for each segmentation method. Partial η^2^ = 0.01 indicates a small effect, η^2^ = 0.06 indicates a medium effect and η^2^ = 0.14 a large effect [30]. In addition, linear regression analysis was performed to assess the relation between normalized volumes and EDSS and average cognition as dependent variables in separate models for each segmentation method, corrected for age and sex. All reported beta values were standardized and R^2^ values were adjusted for the number of variables in each model. *P* values were false discovery rate (FDR) corrected with an alpha of 0.05 and values of *p* < 0.05 were considered statistically significant.

### Validation cohort

In order to verify the robustness of results across different scanners and centers, analyses were repeated for an independent dataset of 125 MS patients. Approval was obtained from the local institutional ethics review board and written informed consent was provided by all participants. Subjects were tested with an extensive battery of neuropsychological tests, which included SRT, SPART, SDMT, Paced Auditory Serial Addition Task (PASAT), WLG and Stroop color test. Z-scores for each test were calculated based on Italian normative data resulting in scores corrected for age, sex and education [31]. Similar to the Amsterdam cognitive data, average cognition was calculated by averaging *Z*-scores from all performed tests.

MRI data were acquired on a 3 Tesla Philips Achieva scanner (Philips Medical Systems, Best, The Netherlands) with an 8-channel head coil. The protocol included a 3D T1-weighted turbo field echo (TFE) sequence (TR 8.1 ms, TE 3.7 ms, flip angle 8°, 1.0 mm sagittal slices, 1.0 × 1.0 mm^2^ in-plane resolution and a 3D FLAIR sequence (TR 4800 ms, TE 291 ms, TI 1650 ms, 1 mm sagittal slices, 0.94 × 0.94 mm^2^ in-plane resolution).

### Data availability

Anonymized data can be shared upon reasonable request from a qualified investigator.

## Results

Table 1 shows the clinical characteristics of the Amsterdam and Verona cohorts. The 329 MS patients of the Amsterdam cohort had a mean age of 48.0 ± 11.0 years, mean symptom duration of 14.5 ± 8.4 years and an EDSS of 3.0 [2.5–4.5]). The 125 MS patients of the validation cohort from Verona had a mean age of 38.8 ± 9.7 years, mean symptom duration of 7.8 ± 6.5 years and median EDSS of 2.0 [1.0–3.0].

### Volumetric agreement with FreeSurfer

**Fig. 1:**
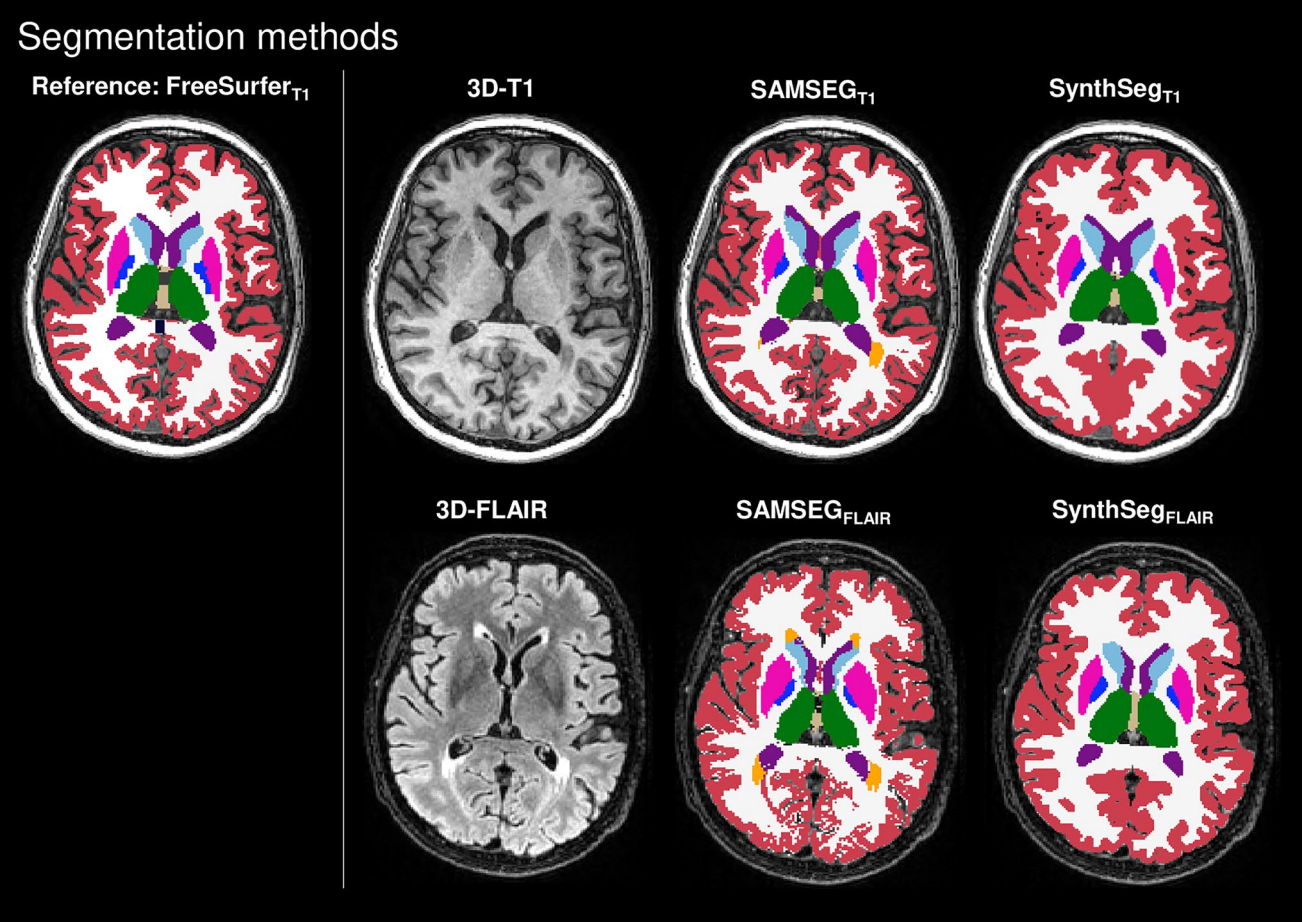
Example of brain segmentation on 3D-T1 and 3D-FLAIR weighted images of an MS subject. FreeSurfer on lesion-filled 3D-T1 was considered the reference method, while SAMSEG and SynthSeg were used to segment unpreprocessed 3D-T1 and 3D-FLAIR weighted images. SAMSEG MS-specific pipeline was used and lesions are displayed in orange

The first step of the analysis was a visual quality check of the different segmentation methods. Figure 1 shows an example of the segmentation outputs of each method. The FreeSurfer_T1_ segmentation showed a large segmentation error for 8 subjects and were excluded from further analyses. SAMSEG and SynthSeg did not show any large segmentation errors. The ICCs for consistency between the non-normalized Freesurfer_T1_ measurements and volumes determined with SAMSEG and SynthSeg on 3D-T1 and 3D-FLAIR are shown in Table 2. All ICC values for brain, ventricle, cortical and DGM volumes were above 0.87 in HC and above 0.90 in MS. For thalamic volumes, the agreement was comparable to the other ICCs for SynthSeg_FLAIR_ (HC: ICC = 0.91, MS: ICC = 0.91), but slightly lower for SAMSEG_FLAIR_ (HC: ICC = 0.82, MS: 0.74).

**Table 2:** ICC between SAMSEG and SynthSeg raw segmentation volumes with reference segmentation of FreeSurfer on 3D-T1

	Brain	Ventricle	Cortex	DGM	Thalamus
HC (n = 76)					
SAMSEG_T1_	0.99	0.98	0.97	0.96	0.88
SynthSeg_T1_	0.99	1.00	0.97	0.98	0.95
SAMSEG_FLAIR_	0.98	0.87	0.93	0.92	0.82
SynthSeg_FLAIR_	0.98	0.96	0.93	0.94	0.91
MS (n = 329)					
SAMSEG_T1_	0.97	0.98	0.97	0.95	0.88
SynthSeg_T1_	0.98	0.99	0.96	0.97	0.94
SAMSEG_FLAIR_	0.98	0.96	0.95	0.90	0.74
SynthSeg_FLAIR_	0.98	0.99	0.96	0.95	0.91

*ICC* intraclass correlation coefficient (consistency, single measures, 95% confidence interval), *DGM* deep gray matter, *HC* healthy controls, *MS* multiple sclerosis;

### Head size normalization

**Fig. 2:**
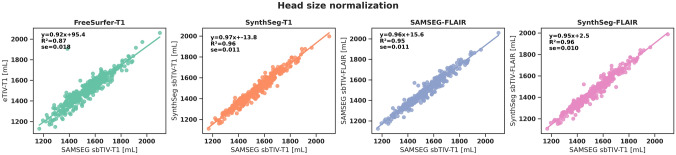
Comparison of head size normalization by between SAMSEG segmentation-based total intracranial volume on T1 (SAMSEG _T1_ sbTIV) versus estimated total intracranial volume on T1 (FreeSurfer_T1_ eTIV), SAMSEG-based sbTIV on FLAIR (SAMSEG_FLAIR_ sbTIV), SynthSeg T1-based sbTIV (SynthSeg_T1_ sbTIV) and on FLAIR (SynthSeg_FLAIR_ sbTIV). Comparisons were performed with linear regression analysis and the equation of the regression lines are shown in the plots

The agreement between head size normalization estimates were analyzed by performing linear regression analyses. SAMSEG_T1_ sbTIV was chosen as the reference method based on previous work (see methods) [28]. The R^2^ between SAMSEG _T1_ sbTIV and SAMSEG _FLAIR_ sbTIV was 0.95 (β = 0.99, se = 0.011), 0.95 for SynthSeg_FLAIR_ sbTIV (β = 0.99, se = 0.011) and 0.96 for SynthSeg_T1_ sbTIV (β = 0.98, se = 0.011) (see Fig. 2). The R^2^ between FreeSurfer_T1_eTIV and SAMSEG_T1_ sbTIV was the lowest from all comparisons (R^2^ = 0.87, β = 0.95, se = 0.018).

### Effect sizes MS versus HC

**Fig. 3:**
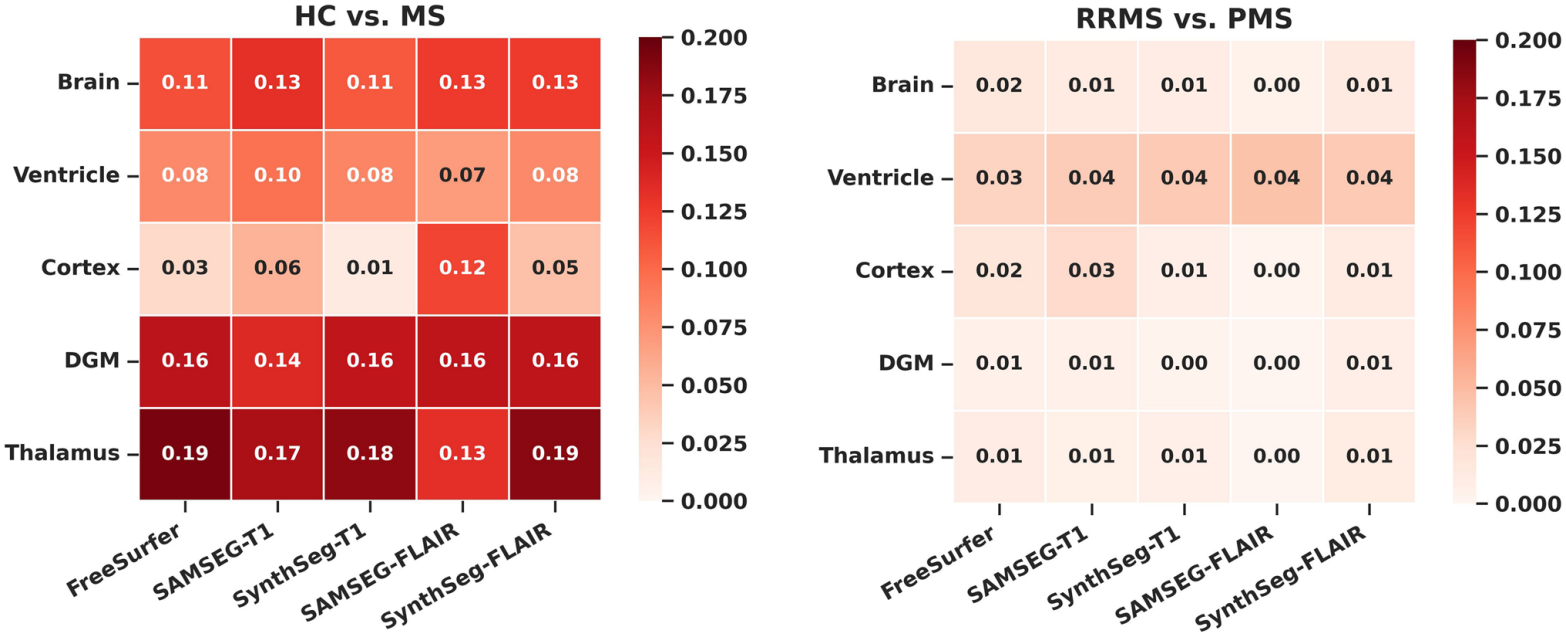
Heatmap of the effect sizes (partial η^2^), comparing normalized volumes between healthy controls vs. MS, and RRMS vs. PMS, corrected for age and sex. Partial η^2^ = 0.01 indicates a small effect, η^2^ = 0.06 indicates a medium effect and η^2^ = 0.14 a large effect

Effect sizes for the differences in normalized MRI volumes for MS vs. HC and RRMS vs. PMS are shown in Fig. 3. Effect sizes between MS and HC were similar for volumes of the brain, ventricles, DGM and thalamus for both FLAIR-based and T1-based methods, with partial η^2^ ranging from 0.07 (ventricles) to 0.19 (thalamus), all *p* < 0.001. For cortical volumes, FreeSurfer_T1_ showed a small effect (η^2^ = 0.03, *p* = 0.001), which was similarly found by SynthSeg_FLAIR_, (η^2^ = 0.05, *p* < 0.001), while SAMSEG_FLAIR_ showed a medium effect (η^2^ = 0.12, *p* < 0.001).

### Effect sizes RRMS versus PMS

Between RRMS and PMS, FreeSurfer_T1_ showed small effect sizes in brain (η^2^ = 0.02, *p* = 0.09), ventricle (η^2^ = 0.03, *p* = 0.005) and cortical volumes (η^2^ = 0.02, *p* = 0.065). These small effects were similarly found with SynthSeg_FLAIR_ (brain: η^2^ = 0.01, *p* = 0.109; ventricle: η^2^ = 0.04, *p* = 0.003; cortex: η^2^ = 0.01, *p* = 0.124), but were absent for SAMSEG_FLAIR_ for brain and cortical volumes, while ventricles were similar (brain: η^2^ = 0.00, *p* = 0.331; ventricle: η^2^ = 0.04, *p* = 0.002; cortex: η^2^ = 0.00, *p* = 0.505).

### Relation between volumes and disability

**Fig. 4:**
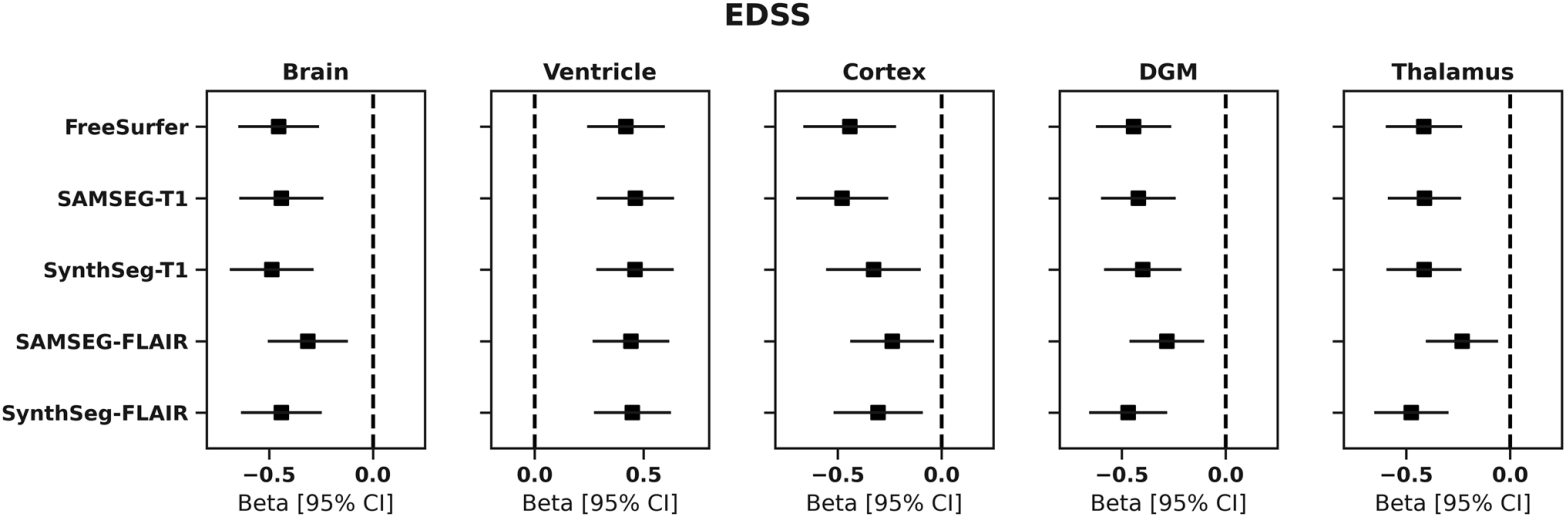
Relationship between EDSS and normalized volume measures calculated by each method. Plots show standardized beta regression coefficients from multivariate linear regression between each volume and EDSS as dependent variable, corrected for age and sex

Standardized regression coefficients of the relationship between EDSS and each individual normalized volume are presented in Fig. 4. There were significant associations between EDSS and brain, ventricle, cortical, DGM an thalamic volumes from all different segmentation methods (p < 0.05). Largest variations in association strength between T1 and FLAIR-based volumes were found for the cortex, where FreeSurfer_T1_ and SAMSEG_T1_ cortical volumes showed the highest association with EDSS (FreeSurfer_T1_:adj. R^2^ = 0.26, std. β = -0.44, *p* < 0.001; SAMSEG_T1_:adj. R^2^ = 0.26, std. β = -0.48, *p* < 0.001), and lower association for SynthSeg_T1_ (adj. R^2^ = 0.24, std. β = -0.33, *p* = 0.005), SynthSeg_FLAIR_ (adj. R^2^ = 0.24, std. β = -0.30, *p* = 0.005) and SAMSEG_FLAIR_ (adj. R^2^ = 0.23, std. β = -0.24, *p* = 0.020).

### Relationship between volumes and average cognition

**Fig. 5:**
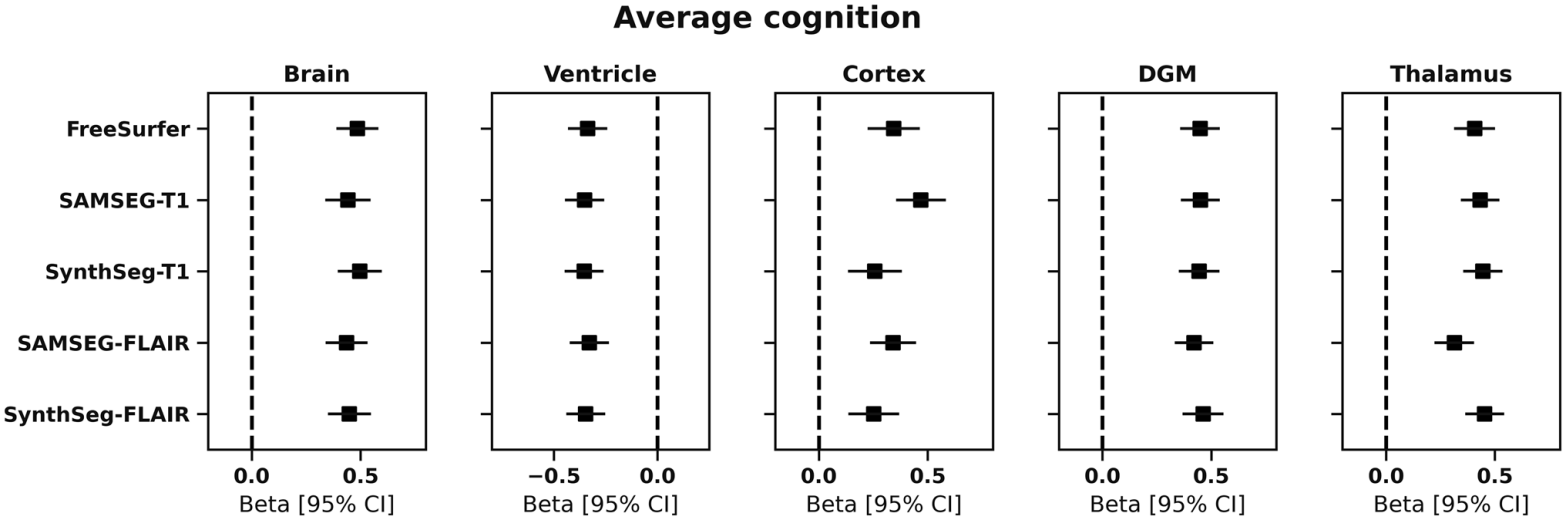
Relationship between average cognition and normalized volume measures calculated by each method. Plots show standardized beta regression coefficients from multivariate linear regression between each volume and average cognition as dependent variable, corrected for age and sex

The association between average cognition and volumes derived from 3D-FLAIR and 3D-T1 are presented in Fig. 5. All methods found an association between average cognition and brain (adj. R^2^ = 0.24–0.29, std. β = 0.44–0.50, *p* < 0.001), ventricle (adj. R^2^ = 0.19–0.21, std. β = -0.33–0.35, *p* < 0.001), cortex (adj. R^2^ = 0.11–0.23, std. β = 0.25–0.47, *p* < 0.001), DGM (adj. R^2^ = 0.27–0.29, std. β = 0.42–0.46, *p* < 0.001) and thalamic volumes (adj. R^2^ = 0.19–0.29, std. β = 0.31–0.45, *p* < 0.001). Again, the largest variations between methods were observed for the cortex.

### Independent validation cohort

Analyses were repeated on an independent validation cohort of 125 participants with MS from Verona (Italy), demographics are shown in Table 1. All ICC values for brain, ventricle, cortical, DGM and thalamic volumes were above 0.86. For the head size normalization strategies, results were highly similar to the Amsterdam results (Supplementary Fig. 1). The R^2^ between SAMSEG _T1_ sbTIV and SAMSEG _FLAIR_ sbTIV was 0.95 (β = 0.95, se = 0.020), 0.95 for SynthSeg_FLAIR_ sbTIV (β = 0.93, se = 0.019) and 0.95 for SynthSeg_T1_ sbTIV (β = 0.95, se = 0.018). The R^2^ between FreeSurfer_T1_ eTIV and SAMSEG _T1_ sbTIV was 0.61 (β = 0.61, se = 0.044), which was lower than the R^2^ observed in the Amsterdam data (R^2^ = 0.87, β = 0.95, se = 0.018).

The association between EDSS, average cognition and volumes derived from 3D-FLAIR and 3D-T1 are presented in Supplementary Fig. 2 and 3. For EDSS and average cognition, all methods had similar associations between these outcome measures and brain, DGM and thalamus volumes (*p* < 0.05) as in the Amsterdam MS cohort. Also similar was the large variation in association strength for the cortical volumes between the different methods and clinical outcomes. While the reference method FreeSurfer_T1_ did not find an association between cortical volume and average cognition (adj. R^2^ = 0.21, std. β = 0.044, *p* = 0.671), SAMSEG_FLAIR_ was the only method that found a relation (adj. R^2^ = 0.27, std. β = 0.25, *p* = 0.010). The correlation between lesion volumes and normalized tissue volumes for both cohorts are shown in Supplementary Fig. 4.

## Discussion

This study investigated the agreement of brain volume measurements on 3D-FLAIR with those on 3D-T1 in a large cross-sectional dataset with MS patients and healthy controls. Our results demonstrated high consistency in the total brain, ventricular and total DGM volumes measured on 3D-FLAIR compared to those measured on standard 3D-T1. However, for cortical and thalamic volumes, the agreement between 3D-FLAIR and 3D-T1 was dependent on the method used, where especially the cortex showed strongest variations for relations to clinical outcome measures. We replicated these findings in an independent validation cohort. Thus, more advanced regional quantifications on 3D-FLAIR require specific choices and further methodological innovation.

A good to excellent consistency was found especially for brain and ventricular volumes [32]. This finding is highly relevant for the adoption of 3D-FLAIR for volume measurements in clinical practice, since whole brain and ventricular volumes are both powerful measures to define, monitor and predict MS severity [2, 33]. For thalamic volumes the agreement was lower using the FLAIR-based methods, especially with SAMSEG. The overall consistency between T1 and FLAIR-based volumes obtained in our study is in agreement with another study using a multi-atlas-based segmentation approach (Geodesic Information Flow, GIF) [10]. However, in that study, only global GM volumes were compared and their method, GIF, was not compared against other standard methods in the field, such as FreeSurfer in this study.

Effect sizes for detecting volume differences in MS compared to controls were stable across methods, which could be expected given the good to excellent relative agreement. Highest effect sizes were found for the thalamus and total DGM, which is consistent with other studies showing the highest atrophy rate in DGM compared to other brain areas [34, 35]. Although we found comparable effect sizes for DGM volumes, another study found systematic differences between DGM segmentation methods (FSL-FIRST, FreeSurfer, GIF and volBrain) compared with manual reference measurements [36]. Moreover, the same study reported reduced accuracy of DGM segmentation in MS versus controls, which could have affected the found effect sizes in the present study since we only used automated segmentation methods. For corticalvolumes, effect sizes between MS and HC were the lowest of all assessed brain structures and showed the largest differences between methods. Cortical segmentation is already a notoriously difficult task on high-resolution 3D-T1 weighted images, with an average variability of 2.5–3% [37] and cortical measurements varying between software [38]. Especially since FLAIR-weighted images are generally not optimized for gray/white matter contrast, a more extensive evaluation of cortical measurements on currently available FLAIR scans is needed.

The most similar correlations for FLAIR-based methods compared to T1-based methods were found for brain and regional volumes with EDSS and average cognition. These correlations were especially highly consistent for the total brain, ventricle, DGM and thalamic volumes. Again, the cortical volumes displayed the largest differences across methods. For example, SAMSEG on 3D-FLAIR falsely detected an association between cortical volumes and average cognition in the validation cohort, while the other T1- and FLAIR-based methods did not show any association. In that regard, SynthSeg cortical segmentation on 3D-FLAIR seems more reliable compared with SAMSEG, although the associations with disability and cognition were less strong compared to FreeSurfer. Since cortical atrophy is an important outcome measure for cognition and has been shown to be clinically predictive for cognitive decline [18], further validation of 3D-FLAIR derived cortical volumes is warranted before using FLAIR derived cortical volumes in relation to cognitive outcomes.

Although 3D-FLAIR sequences are currently recommended for MS diagnosis and monitoring [7], clinical legacy datasets mostly contain 2D-FLAIR scans. Retrospective analysis of these large clinical databases would enable retrospective studies with high sample sizes to gain more insight in MS. SynthSeg has originally been developed with the aim to provide accurate segmentation on low resolution scans of any contrast type, so future work should investigate the segmentation accuracy on 2D-FLAIR compared with 3D-FLAIR. SAMSEG may also be a promising candidate to segment 2D-FLAIR scans, but was designed and validated mostly on high resolution images in MS [11]. The advantage of SAMSEG for application to clinical legacy data is that it currently has a longitudinal pipeline, while SynthSeg is a cross-sectional method.

This study is not without limitations. First, although we replicated our findings in an independent validation cohort from a different center, all data were acquired on 3.0 T MRI scanners. Since clinical MRI scanners often have lower field strength, findings should be replicated on lower field strengths and between scanners as well [39]. Second, the outcomes of this study rely on the surface-based FreeSurfer stream as the reference segmentation. Although FreeSurfer is a well-established research method, manual reference segmentations still remain the golden standard, which was not feasible to create for the large data set used in this study. Third, only cross-sectional correlations of FLAIR images with clinical outcome measures were assessed, but the longitudinal relations of these segmentation methods on FLAIR is unknown. This is especially relevant for facilitating clinical implementation of these techniques since brain atrophy rates are useful measures for assessing disease evolution and treatment response [19]. In addition, in real-world clinical settings, patients are scanned on different scanners and protocols are constantly updated over time. Therefore, the effect of different scanners and protocols on longitudinal measurements should be a topic of future study as well.

## Conclusion

Brain volumes segmented on 3D-FLAIR with SynthSeg and SAMSEG show a good to excellent agreement with FreeSurfer-derived 3D-T1 segmentation in MS, especially for total brain and ventricular volumes. Lower volume in MS vs. HC on 3D-FLAIR was relevant for disability and cognitive dysfunction, but effect sizes depended on the segmentation method that was used. While agreement of total DGM, total brain and ventricular segmentation was relatively good, cortical segmentation remains especially difficult, which could be the focus of further improvement of FLAIR-based segmentation methods.

### Supplementary Information

Below is the link to the electronic supplementary material.

Supplementary file1 (DOCX 355 KB)
