# Supplementary material for: Feasibility of detecting atrophy relevant for disability and cognition in multiple sclerosis using 3D-FLAIR
Source: J Neurol. 2023 Jul 19;270(11):5201–10. doi: 10.1007/s00415-023-11870-4 (PMC10576669; doi:10.1007/s00415-023-11870-4)
Supplement: Supplementary file 1 — Supplementary file1 (DOCX 355 KB) [file 415_2023_11870_MOESM1_ESM.docx]

**Supplementary materials**

**Table 1.** ICC for consistency between SAMSEG and SynthSeg volumes with reference segmentation of FreeSurfer on 3D-T1 for the Verona validation cohort.

| **Verona** | | **Brain** | **Ventricle** | **Cortex** | **DGM** | **Thalamus** |
| --- | --- | --- | --- | --- | --- | --- |
| **MS (n=125)** | |  |  |  |  |  |
|  | SAMSEG T1 | 0.97 | 0.97 | 0.95 | 0.95 | 0.88 |
|  | SynthSeg T1 | 0.99 | 0.98 | 0.96 | 0.98 | 0.96 |
|  | SAMSEG FLAIR | 0.99 | 0.93 | 0.93 | 0.94 | 0.86 |
|  | SynthSeg FLAIR | 0.99 | 0.98 | 0.94 | 0.97 | 0.94 |

*Abbreviations: ICC = intraclass correlation coefficient; DGM = deep gray matter; HC = healthy controls; MS = multiple sclerosis;*


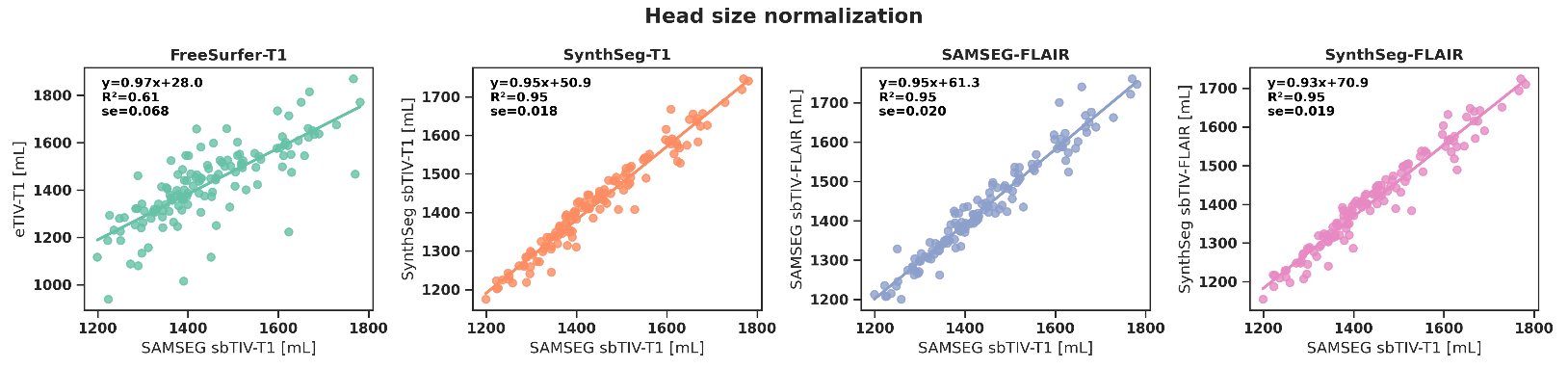


**Figure 1.** Comparison of head size normalization by between SAMSEG segmentation-based total intracranial volume on T1 (SAMSEG sbTIV_T1_) versus estimated total intracranial volume on T1 (eTIV_T1_), SAMSEG-based sbTIV on FLAIR (sbTIV_FLAIR_), SynthSeg-based sbTIV_T1_ and sbTIV_FLAIR_. Comparisons were performed with linear regression analysis and the equation of the regression lines are shown in the plots. Results shown are for the Verona cohort (n=125 MS patients).


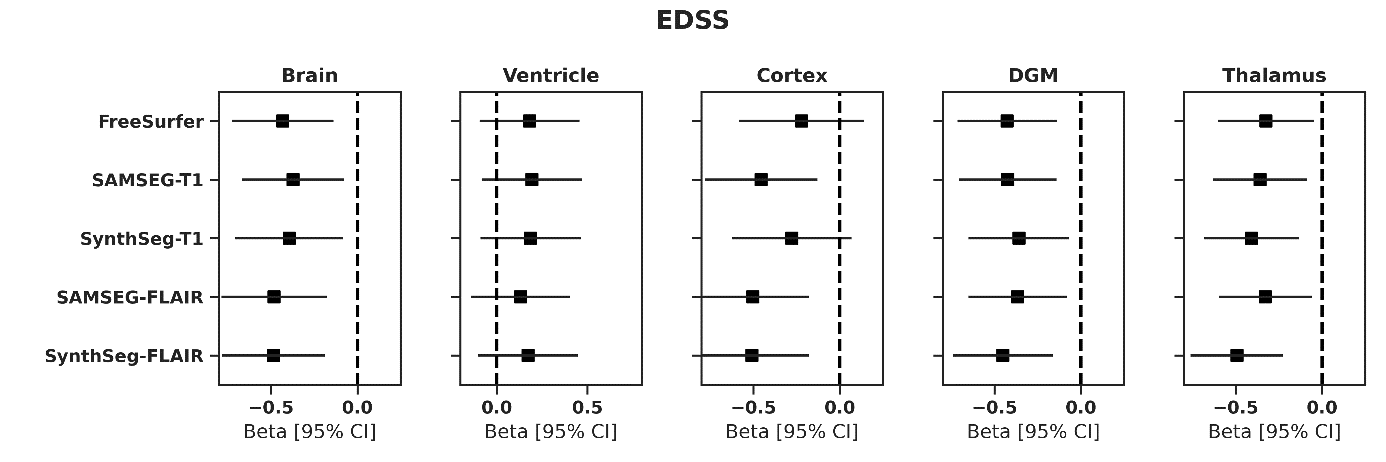


**Figure 2.** Relationship between EDSS and normalized volume measures calculated by each method in Verona cohort (n=125 MS patients). Plots show standardized beta regression coefficients from multivariate linear regression between each volume and EDSS as dependent variable, corrected for age and sex.


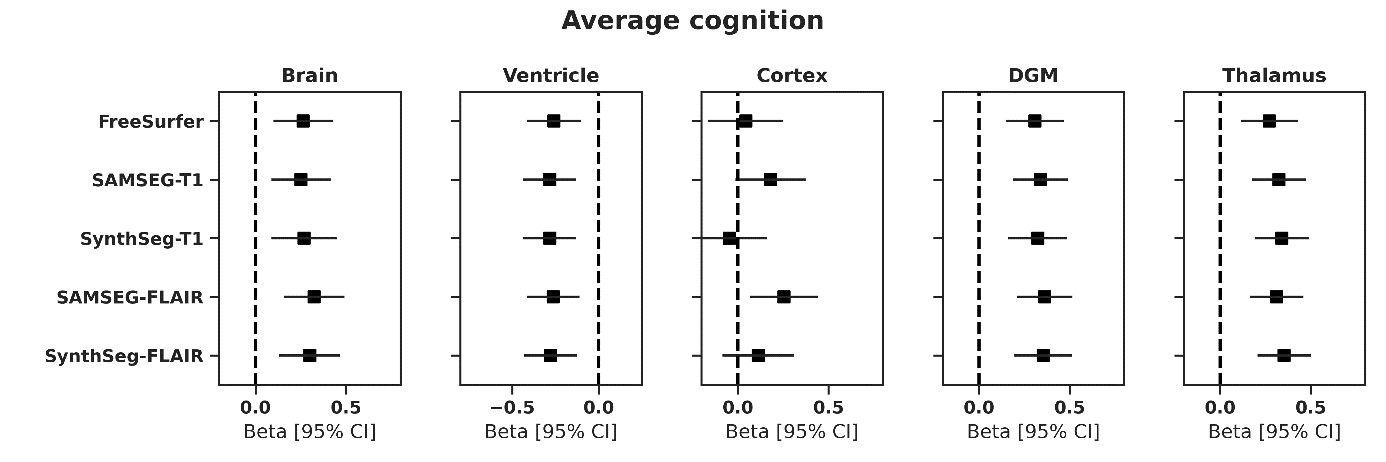


**Figure 3.** Relationship between average cognition and normalized volume measures calculated by each method in Verona cohort (n=125 MS patients). Plots show standardized beta regression coefficients from multivariate linear regression between each volume and average cognition as dependent variable, corrected for age and sex.


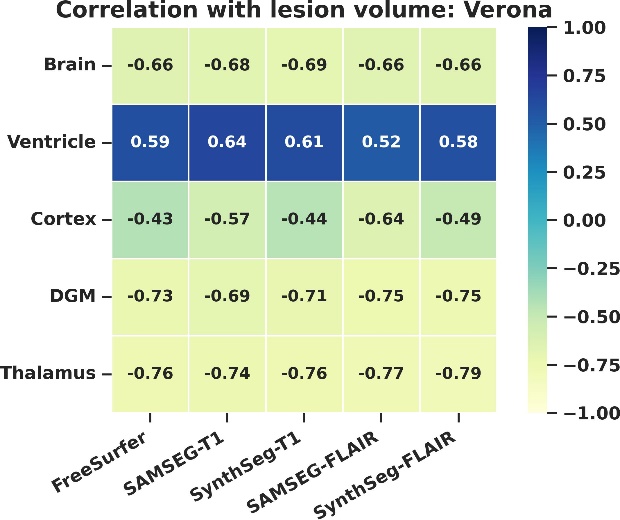


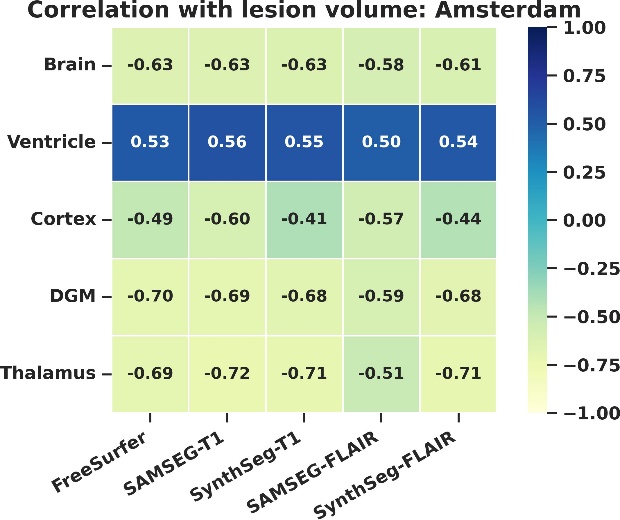


**Figure 4.** Pearson correlation between lesion volume (log-transformed) and normalized volumes.
